# A complex histopathological challenge: suspicion of an osteoblastoma-like osteosarcoma arising from the second thoracic vertebra in a cat

**DOI:** 10.1186/s12917-020-02596-w

**Published:** 2020-10-07

**Authors:** Felix Giebels, Franck Forterre, Simona Vincenti, Urs Geissbuehler, Monika M. Welle, Roy Pool, Sabina Soldati, Arianna Maiolini

**Affiliations:** 1 Department of Clinical Veterinary Medicine, Division of Clinical Neurology, Vetsuisse Faculty of Bern, University of Bern, Bern, Switzerland; 2 Department of Clinical Veterinary Medicine, Division of Small Animal Surgery, Vetsuisse Faculty of Bern, University of Bern, Bern, Switzerland; 3 Department of Clinical Veterinary Medicine, Division of Clinical Radiology, Vetsuisse Faculty of Bern, University of Bern, Bern, Switzerland; 4 Institute of Animal Pathology, Vetsuisse Faculty of Bern, University of Bern, Bern, Switzerland; 5 Department of Veterinary Pathobiology, Veterinary Medicine and Biomedical Sciences, Texas A & M University, College Station, TX USA; 6 Department of Veterinary Medicine, University of Milan, Lodi, Italy; 7 Mouse & Animal Pathology Lab (MAPLab), Fondazione UniMi, Milan, Italy

**Keywords:** Vertebral column, Neoplasia, Spinal cord, Feline, Bone tumour

## Abstract

**Background:**

Reports of osteoblastic tumours are limited to a few case reports in veterinary medicine. Osteoblastoma-like osteosarcoma has been accepted by the World Health Organization as an intermediate form between an osteosarcoma and osteoblastoma. This type of tumour indicates an osteosarcoma, that may resemble osteoblastoma clinically, histologically, and radiologically and have the capability for metastasis. Osteoblastoma-like osteosarcoma has not been described in veterinary medicine so far.

**Case presentation:**

An eight-year old cat was presented due to progressive ataxia and paraparesis of the pelvic limbs. Imaging confirmed a well-defined, extradural mass originating from the spinous process of the second thoracic vertebra (T2) leading to severe compression of the spinal cord. Decompressive cytoreduction was achieved by removal of the mass after dorsal laminectomy of T1. After recovering from an acute worsening 3.5 weeks after surgery, the cat had an improved neurological status and the dorsal compression was resolved at follow-up 8 months later. A focal contrast enhancing lesion was still evident at the base of T2 spinous process and lung metastasis was additionally suspected. Based on histopathological, radiographic, and clinical features, an “osteoblastoma-like osteosarcoma” was suspected.

**Conclusions:**

To the best of our knowledge, this is the first description of this tumour in veterinary medicine. In addition, this case report highlights the difficulty in the diagnosis and definition of osseous neoplasia in cats and provides a literature review.

## Background

Osteosarcoma represents the most common bone tumour in cats, encompassing 70–80% of these neoplasms [1–6]. In one study investigating 85 feline spinal tumours, osteosarcoma was the most common vertebral tumour and represented the second largest fraction of all diagnosed tumours [7].

In contrast, reports of osteoblastic tumours in the veterinary literature are limited, and include lesions reported in three cats [4, 8], two dogs [9], mice [10], a pony [11] and a hedgehog [12]. Similarly, in human medicine, osteoblastoma is a very rare primary bone neoplasia accounting for less than 1 % of all bone forming tumours [13, 14]. In cats, Liu and others [4] identified an osteoid osteoma in a thoracic vertebral body.

Interestingly, in human literature, intermediate types have been described that combines features of both, osteosarcoma and osteoblastoma and led to a complex definition or redefinition of different subtypes of osteosarcoma and osteoblastoma. Table 1 gives a short overview over the described subtypes.

**Table 1:** Overview of the described intermediate types between osteosarcoma and osteoblastoma in human literature

Source	Definition
Schajowicz and Lemos (1976) [15]	*Malignant osteoblastoma*
Mirra and others (1976) [16]	*Pseudomalignant osteoblastoma*
Revell and Scholtz (1979) [17]	*Aggressive osteoblastoma*
Dorfman and Weiss (1984) [18]	Subclassification in: *1. low-grade osteoblastoma-like osteosarcomas* *2. pseudomalignant osteoblastoma* *3. malignant transformation of initially benign osteoblastoma to a high-grade malignant osteosarcoma* *4. locally aggressive/ malignant osteoblastoma*
Bertoni and others (1985) [19]	*Osteosarcoma resembling osteoblastoma*
Bertoni and others (1993) [20]	*Osteoblastoma-like osteosarcoma*
Bertoni and others (1993) [21]	*Osteoblastoma with cartilaginous matrix*
De Oliveira and others (2007) [22]	*Atypical osteoblastoma*

Osteoblastoma-like osteosarcoma (OBLOS) has been defined in human literature as a malignant tumour, that share features between both, osteosarcoma and osteoblastoma and have the potential for metastasising [19, 23]. The term “*aggressive osteoblastoma*” was first introduced into veterinary literature describing a vertebral neoplasia in a cat by Kim et al. [24]. Aggressive osteoblastoma invades adjacent tissue and recurs but, in contrast to OBLOS, does not metastasize [25]. Although malignant transformation of osteoblastomas with pulmonary metastases have been infrequently reported in human medicine [26–29], this has not been described in veterinary medicine.

In this case report, we describe an osseous neoplasia arising from a thoracic vertebra of a cat that histologically is compatible with an osteoblastoma. However, the clinical and radiological behaviour suggest that the mass represents an intermediate form of osteoblastoma and osteosarcoma previously described in human medicine (i. e. OBLOS, “osteosarcoma resembling osteoblastoma” [19, 20]) but still novel in veterinary medicine and add important information to the sparse literature on osseous neoplasia existing for the feline patient.

## Case presentation

### Clinical history

An eight-year old female spayed domestic short hair cat was presented to the Small Animal Clinic of the Vetsuisse Faculty of the University of Bern, Switzerland, due to progressive gait abnormalities of both pelvic limbs. The first clinical sign, consisting of frequent stretching of the pelvic limbs, has been intermittently observed by the owner over the last 2 months prior to presentation. One week prior to presentation the cat acutely worsened, was reluctant to jump and to climb stairs and seemed to be painful. Additionally, an increased water-intake and periuria have been observed by the owner.

### Clinical findings and investigation

**Fig. 1:**
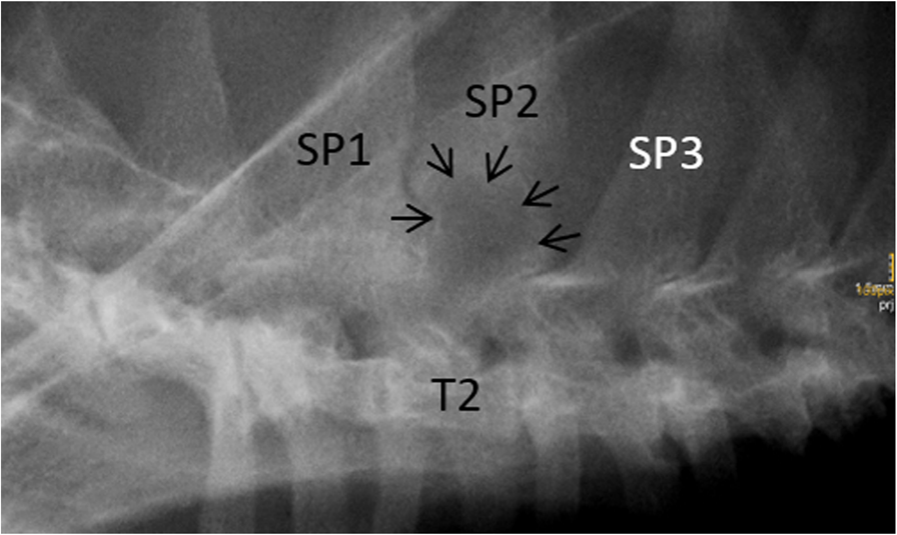
Right to left-lateral radiograph cranial aspect of the thoracic vertebral column. A quite well-defined radiolucent lesion at the level of the base of the spinous process (SP) of the 2nd thoracic vertebra (T2) is visible (arrows). Note the absent opacity originating from the dorsal lamina of T2 and of the facet joints cranial to the lesion. The base of the spinous process of T1 and the area dorsal to the lesion appear diffusely sclerotic

**Fig. 2:**
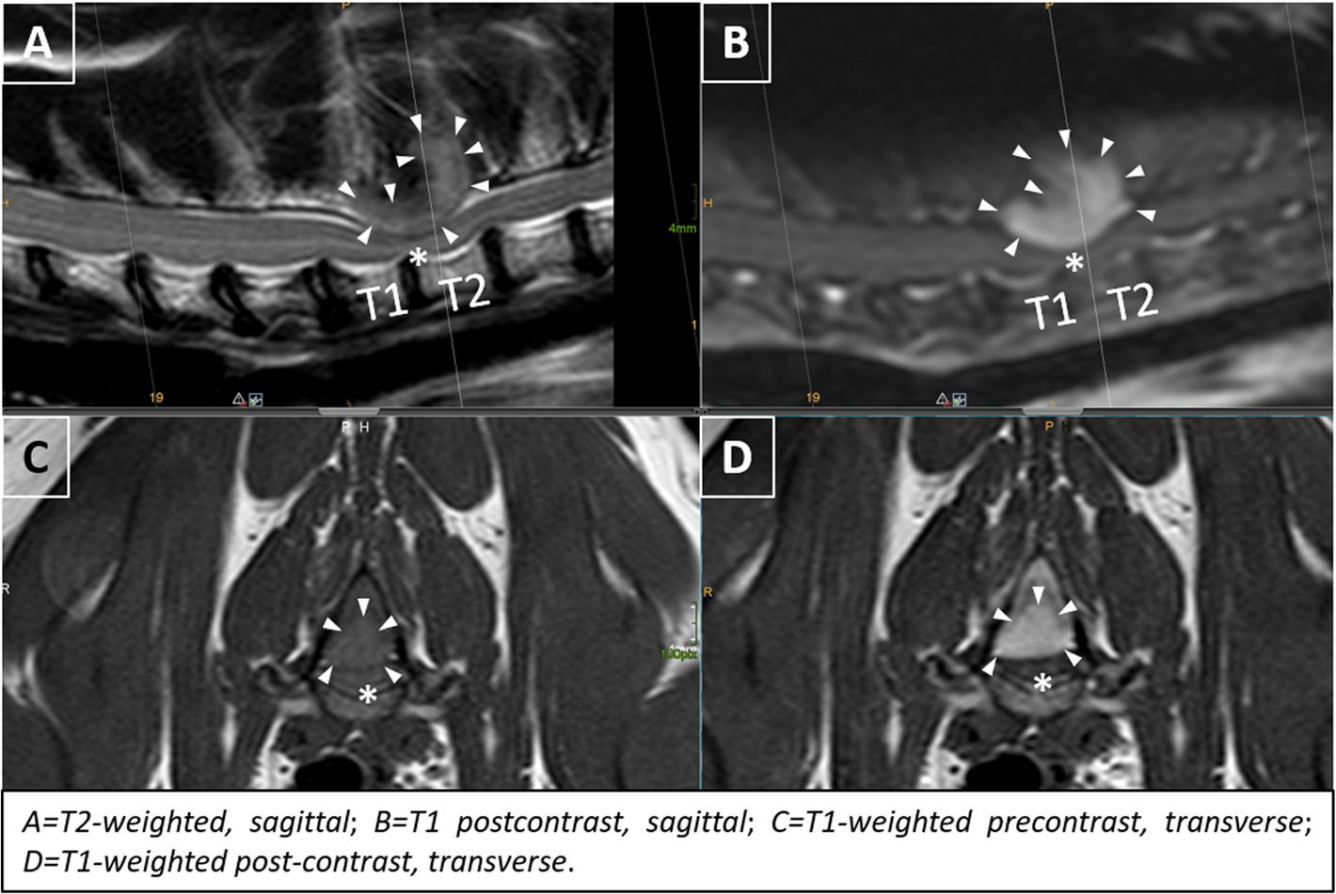
MRI at the level of T1/2. An extramedullary lesion (arrowheads) invading the ventral medullary space of the spinous process of T2 with homogenous contrast enhancement (**b**, **d**) is visibile. Medullary invasion is best outlined after contrast injection (**b**, **d**). A severe dorsal compression of the spinal cord (*) exists

**Fig. 3:**
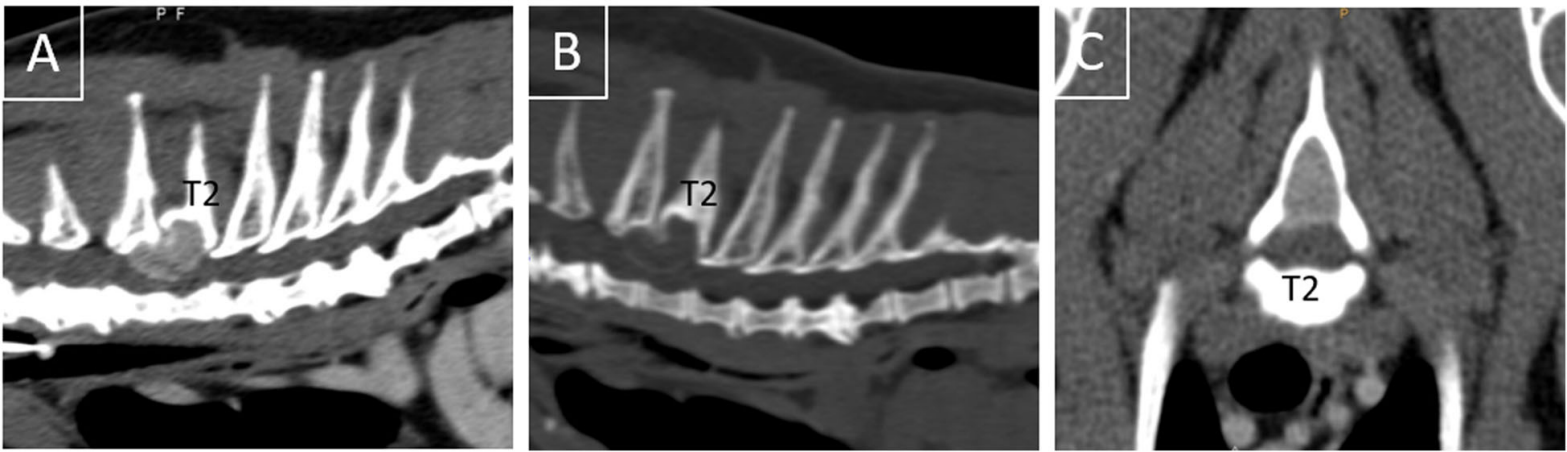
Sagittal reconstruction (**a, b**) and transverse (**c**) CT-images of the cranial thoracic vertebral column presented in soft tissue- (**a, c**) and bone window post contrast (**b**). A thin, hyperdense line separates the bilobed lesion ventrally from the underlying spinal cord and is the remnant of the ventral and cranial displaced dorsal lamina of T2 (**b,c**). Note the thinning and deformation of the cortex of the spinous process of T2 and its widening of the medullary cavity at the base (**c**)

At the time of presentation, vital parameters were within normal limits. Neurological examination revealed mild ambulatory paraparesis, moderate pelvic limb proprioceptive ataxia, and reduced proprioceptive positioning and hopping response in both pelvic limbs. The remaining neurological examination was unremarkable and no spinal hyperesthesia could be detected on palpation. Neurological findings were consistent with a lesion within the third thoracic and third lumbar spinal cord segments. Initial differential diagnoses included neoplasia, intervertebral disk protrusion and an inflammatory process. Haematology and serum biochemistry revealed mild changes, including eosinophilia (1.94 × 10^9^/l; range: 0–1.5 × 10^9^/l), and an elevated aspartate transaminase (117 IU; range: 12–61) and creatinine kinase (5812 IU; range: 0–596) activity. Serological testing for *feline immunodeficiency virus* and *feline leukaemia virus* were negative. Thoracic radiographs revealed no abnormalities of the intrathoracic organs, although a quite well defined, round geographic radiolucent lesion was identified at the base of the spinous process of the second thoracic vertebra (T2) expanding into the area of the vertebral canal (Fig. 1). MRI using a 1.0 Tesla open permanent magnet (Philips HFO Panorama, Philips Medical Systems, PC Best, Netherlands) of the thoracolumbar area revealed an extramedullary space occupying lesion at the level of T1/2, causing a severe dorsal compression and deformation of the spinal cord (Fig. 2). The lesion was slightly heterogeneous and almost isointense to the spinal cord on T1- and T2-weighted and hyperintense on T2*- and STIR- sequences. The epicenter of the lesion was suspected to be at the level of the dorsal lamina of T2. For a better description of bony involvement and to rule out metastases, the MRI was immediately followed by a CT-examination of the thorax and abdomen. In CT, the above described lesion had a soft tissue density (HU 60–65) (Fig. 3). The mass showed severe and relatively homogeneous contrast enhancement.

The final radiographic diagnosis was an expansile extramedullary space occupying mass centered in the cancellous bone of the base of the dorsal spinous process of T2 causing severe compression of the spinal cord from dorsal and dislocation and deformation of the caudodorsal lamina of T1. No signs of metastatic disease were detected. Cerebrospinal fluid was collected from the cerebellomedullary cistern and its analysis was unremarkable.

## Differential diagnosis

Considering the signalment, the clinical and imaging presentation, the main differential diagnosis was a vertebral neoplasia, i. e. round cell tumour, osteoblastoma or osteosarcoma.

## Treatment

The cat was discharged with gabapentin (5 mg/kg TID po) and restriction of exercise was recommended. A surgical exploration was suggested and performed fortnight after diagnosis.

**Fig. 4:**
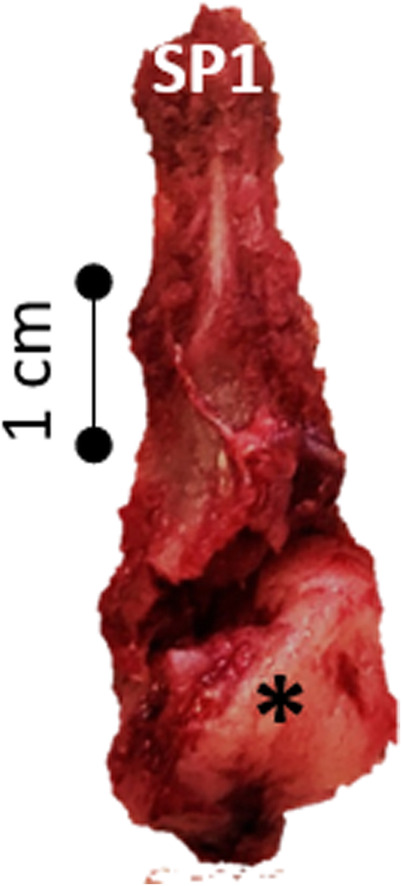
Caudal view on the resected spinous process of T1 (SP1). Note the enlargement of the medullary cavity at the spinous process´ base and the brownish and smooth surface of the tumour (*)

During surgery, a large dorsal laminectomy of T1 (modified Funkquist type B) was performed. A white, solid bulging mass originating from the lamina of T2 was identified on the excised bony segment (see Fig. 4). After its resection, the underlying spinal cord showed a dorsal indention at the level of T2. Based on the intraoperative findings, the mass seemed to be macroscopically completely removed, therefore a caudal extension of the laminectomy over T2 was not performed. The cat recovered uneventfully, and the early post-surgical treatment included fentanyl-CRI (5 μl/kg/h), gabapentin (5 mg/kg TID po), prednisolone (1 mg/kg SID po) and omeprazole (1 mg/kg BID iv). After the first 24 h following surgery, fentanyl was substituted with buprenorphine (.01 mg/kg TID iv).

## Histopathological findings

For histological examination the resected material was fixed in 10% buffered formalin, decalcified (*Rapid Decalcifier*, J.T. Baker®), routinely processed, and stained with hematoxylin & eosin and van Gieson’s stain.

**Fig. 5:**
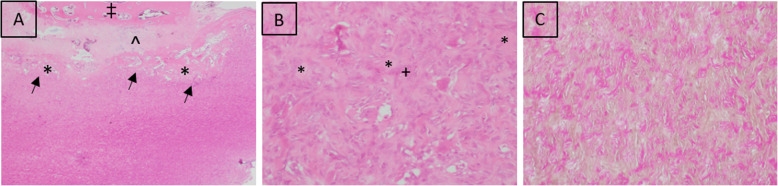
Histological presentation, Hematoxylin & Eosin: **a** The expansively growing neoplastic mass develops (arrows) within the intertrabecular spaces of the irregularly arranged woven bone (*), which is attached to the fibrous periosteum in which chondrocytes (^) and osteoblasts are present. ‡ = base of the spinous process of T1. **b** Close to the periosteum the tumour is composed of larger lakes of amorphous osteoid (*) surrounded by polygonal osteoblasts and occasional multinucleated osteoclasts (+) 400x. **c** Van Gieson: Mass further away from the periosteum. Osteoid production was less and short fascicles of spindle cells or polygonal cells (yellow) surround small islets of osteoid (red) 100x

At first, a periosteal osteosarcoma was diagnosed. This diagnosis was based on the periosteal covering of the tumour’s surface (Fig. 5a).

After discussion of the case in the context of the diagnostic imaging, histology was re-evaluated by a pathologist specialized in bone pathology. Histopathologically, this specimen was an ovoid, low-grade tumor resembling neoplastic cells of osteogenic origin that were spindled and polygonal cells having mild anaplastic features. Tumor cells that exhibit few mitotic figures form and entrap themselves in random microscopic deposits of osteoid matrix. Tumor expansion toward the spinal canal has induced osteoclastic remodeling and removal of the bony support for the periosteal covered surface. Neoplastic cells revealed varying patterns within the mass; close to the periosteum the mass was composed of larger lakes of osteoid surrounded by polygonal osteoblasts and occasional multinucleated osteoclasts (Fig. 5b). Further, apart from the periosteum, osteoid production was less, and the tumour was composed of short fascicles of neoplastic spindle cells surrounding small islets of osteoid matrix; in addition, few polygonal cells, resembling osteoblasts and few osteoclasts were present (Fig. 5c).

Based on the histopathological findings, an osteoblastoma was diagnosed.

## Outcome and follow-up

**Fig. 6:**
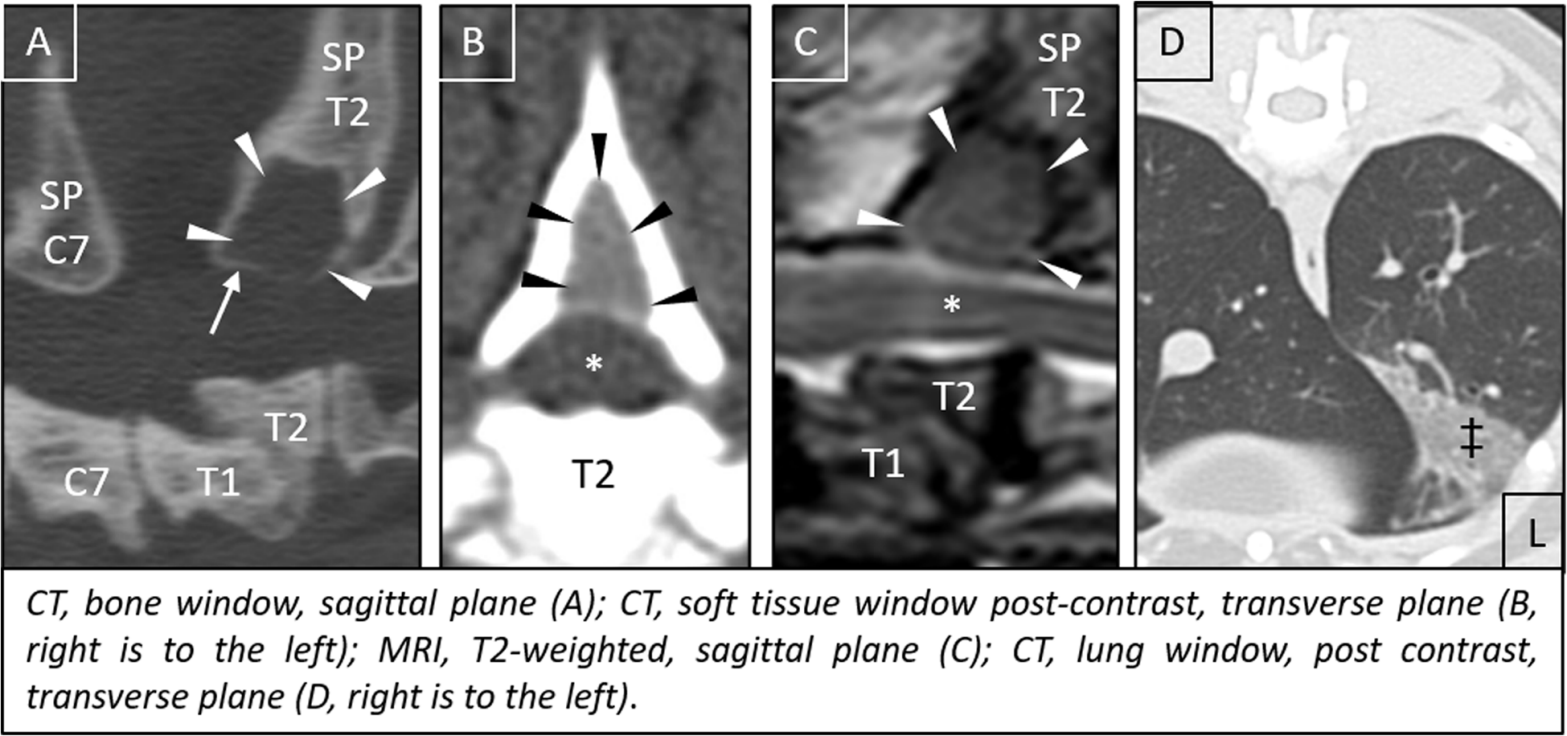
Follow-up diagnostic imaging. Arrowheads outline a well demarcated, hypodense bone lesion at the base of the spinous process (SP) of the 2nd thoracic vertebra (T2), which is ventrally delineated by a bony rim (arrow, **a**). Note the dorsal subluxation and synostosis between the 1st (T1) and 2nd (T2) thoracic vertebra. The lesion takes up contrast homogenously (**b**). Spinal cord compression (*) is no longer existing (**c**). Lung changes were present in the left (L) caudal lung lobe (‡, **d**)

The cat was discharged 2 days after surgery with an ambulatory paraparesis and proprioceptive ataxia in both pelvic limbs. Further medical treatment consisted in gabapentin (5 mg/kg TID po for 2 weeks, thereafter slow withdrawal over 2 weeks), prednisolone (1 mg/kg SID po for 5 days, thereafter slow withdrawal over 15 days) and omeprazole (1 mg/kg BID po during prednisolone-treatment). Strict cage rest for the first weeks after surgery and physiotherapy were recommended. At the first follow-up 2 weeks after surgery, the cat experienced a clear improvement of the neurological signs, showing only a mild ataxia in both pelvic limbs. Due to the temperament of the cat, physiotherapy could not be performed. Nevertheless, after initial improvement, the cat suffered an acute transient deterioration 3.5 weeks after surgery which improved with strict rest. At the clinical and imaging follow-up 8 months after surgery, the cat had only a mild ataxia in the pelvic limbs. CT revealed a well-defined, in respect to the spinal cord isodense lesion ventrally demarcated by a hyperdense rim within the base of the spinous process of T2, that was homogenously contrast enhancing, indicating recurrence of the tumour (Fig. 6a, b). Furthermore, a dorsal T1/2 vertebral luxation with synostosis was diagnosed. Nevertheless, MRI showed a resolution of the dorsal compression of the spinal cord (Fig. 6c). Additionally, a triangular ground glass opacity lung lesion ventrally within the left caudal lobe was visible. (Fig. 6d).

Due to the current good quality of the cat’s life, the owner denied further workup of the pulmonary changes, as well as any additional treatment. As metastatic lung disease could not be excluded, a histopathological re-evaluation including immunohistochemistry was performed. Primary antibodies used were COX-2, to differentiate osteoblastoma from low-grade osteosarcoma and Collagen I, to confirm osteoid matrix production and therefore osseous origin of the neoplasia. Heat induced epitope retrieval (devax and HIER Buffer H at pH 9, Thermoscientific) for unmasking antigens was used. Negative immunohistochemical controls for each sample were prepared by omitting the primary antibody. For each primary antibody, a known positive control section was included in each immunolabeling assay.

**Fig. 7:**
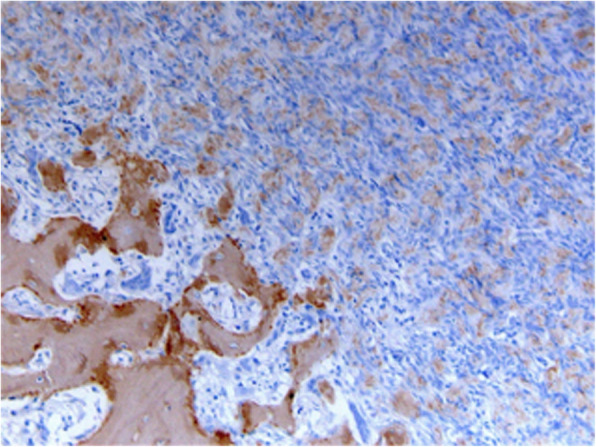
Immunohistochemistry (IHC) for Collagen I. Diffuse positivity evidenced by the brown colour of the reactive woven bone and the extracellular osteoid matrix of the tumour, 200x. Hematoxylin was used for counterstaining

COX-2 immunostaining revealed strong positivity of few neoplastic cells entrapped in a focal area of woven bone, while the rest of the neoplastic cells were negative. Collagen I immunostaining was diffusely positive within the cortical bone next to the tumoral mass, the reactive woven bone and the extracellular osteoid matrix of the tumour (Fig. 7). In summary, considering histopathological and diagnostic imaging findings, the inconclusive immunoreactivity of the sample and the possible lung metastasis, an OBLOS arising from T2 was suspected, although it could not be confirmed.

At the date of submission (i. e. nearly 23 months after initial presentation) the cat was still alive and showed a good quality of life.

## Discussion

Osteosarcoma is the most common vertebral tumour in the cat. In contrast, veterinary reports of osteoblastic tumours are restricted to a few case reports [8–12, 24, 30].

Definitive diagnosis of primary bone tumours is challenging. These difficulties are highlighted in this case report, in which initially, based on the histopathological findings, a periosteal osteosarcoma was diagnosed. This diagnosis was based on the periosteal covering of the mass. The first diagnosis led to some concerns, since based on imaging, the lesion seemed to originate from the cancellous bone of T2, rather than its surface. Therefore, a pathologist specialized in bone pathology was consulted for a second opinion, who diagnosed finally an osteoblastoma arising from the cancellous bone of T2 with slow expansile growth that, in turn, has been resulted in encroachment of the border of the spinous process into the spinal canal causing clinical signs.

After a lung metastasis was considered during the follow up, a further re-evaluation of the specimen via immunohistochemistry was performed, since an osteoblastoma would not be compatible with metastatic behaviour. Collagen 1-immunostaining confirmed the production of osteoid matrix by the tumoral cells, and therefore the osseous origin, being Collagen I the main fibrillar component of bone extracellular matrix. In human medicine, COX-2 positivity has been used to distinguish between aggressive osteoblastoma and low-grade osteosarcoma, being the first COX-2 positive and the second COX-2 negative [31]. Kim et al. in 2017 [24] used this immunohistochemical method to diagnose an aggressive osteoblastoma in a cervical vertebra in an Angora cat. Unfortunately, in our case immunohistochemistry failed to result in a definitive diagnosis. COX-2 immunostaining revealed only few although strongly positive neoplastic cells entrapped in a focal area of woven bone, while the rest of the neoplastic cells were negative. Since the tissue underwent decalcification, we hypothesized that the lack of diffuse positivity could be the consequence of an altered immunohistochemical reactivity of the tissue leading to a false negative result in most of the sample. Indeed, in the presented case, a rapid decalcifying solution containing hydrochloric acid was used. Rapid decalcifiers are detrimental for antigenicity, but have the advantage of a fast sample processing, leading to a quicker histological interpretation and therefore preferred in clinical settings. EDTA decalcifying solution has a long decalcification time but preserves antigens well and should be preferred in cases in which immunohistochemistry is planned [32]. Unfortunately, no detailed information about decalcification process and IHC methods are available in the report of Kim et al. [28].

Based on the histological findings, the imaging features and the potential metastatic behaviour, an OBLOS was considered to be the most likely diagnosis. According to the grading by Gambarotti et al. [23], the World Health Organization in its *4th WHO classification of tumours of soft tissue and bone* (2013) listed OBLOS within the group of conventional (high-grade) osteosarcoma. Nevertheless, Gambarotti et al. [23] found in his study OBLOSs that could be subtyped as both, low-grade and high-grade-osteosarcomas and concluded, that the presence of histological areas of conventional (high-grade) osteosarcomas are the only predictor for aggressiveness. Osteoblastoma, aggressive osteoblastoma and OBLOS can share similar histological features, therefore they can be indistinguishable on biopsies. Even if recurrence is described in all three types of tumours, only OBLOS have been shown to metastasize. Permeation of the surrounding bone is an important feature of osteosarcoma [19] and, thus, an often mentioned point in borderline osteoblastic tumours [19, 20, 33]. The histopathological analysis of the specimens in our case does not confirm permeation of the bone of T1 and radiologic analysis does not reveal aggressive bone lesions of T2. This would support the diagnosis of osteoblastoma. However, the clinical follow up, suggesting recurrence with pulmonary changes not excluding metastasis, together with the unreliable COX-2 immunostaining made an OBLOS a more likely differential diagnosis.

No other potential neoplastic lesions were detected on the whole body CT-scan at the time of first presentation; thus, it was presumed that the lung lesion in the follow-up CT scan 9 months later might represent a metastasis of the vertebral bone lesion. The diagnosis of metastatic lung disease tumours is more challenging in cats compared to dogs [34]. In contrary to dogs, multiple nodular lung metastases seem to be rare in cats. Number, distribution, density, size, demarcation, localisation, and contrast uptake of metastatic lung lesions in cats are, in contrary to primary lung neoplasia - especially the adenocarcinoma [35]- hardly documented in the literature. It is reported, that lung metastasis are often less well delineated in cats, similar to the lung nodule in the case presented here [34].

Reasons for the difficulties in diagnosing primary bone tumours are on one side the overlap in histopathological and diagnostic imaging features among the different types (i. e. osteoblastoma and osteoid osteoma, low-grade osteosarcoma and aggressive osteoblastoma) [9, 19, 20, 22, 31, 36–38] and on the other side the ability of malignant transformation [27, 29, 38–40]. In human medicine, this results in the definition or redefinition of different subtypes of osteosarcoma and osteoblastoma [18, 22, 41].

Rosado et al. in 2000 [38] provided a review about the difficulty in differentiating osteoblastoma from osteosarcoma for the human medicine. According to the authors, the tumour’s mitotic activity and its permeation of adjacent tissue are the most important criteria: osteoblastoma reveals no mitotic activity and does not permeate the surrounding tissue whereas osteosarcoma does. Nevertheless, they confirmed the existence of a “*biologically intermediate or indeterminate group*”. Dorfman and Weiss [18] proposed a classification of human borderline osteoblastic tumours into 4 categories (see Table 1). In the cat presented here, OBLOS was considered to be a differential diagnosis to aggressive osteoblastoma. In human literature, the term OBLOS indicates an osteosarcoma, that may resemble osteoblastoma clinically, histologically and radiologically and have the capability for metastasis. However, conflicting information concerning the capacity to metastasize of this type of tumour exists [18, 19, 23–25, 27, 33, 40, 42–46].

In line with our findings, osteoblastoma usually presents as a well-defined, rounded, lytic, radiolucent lesion originating in the medullary part of the bone with varying amounts of surrounding sclerosis in radiographic analysis [38, 47]. In contrast, osteosarcomas are typically destructive with an accompanying periosteal reaction [38]. Furthermore, Rosado et al. in 2000 [38] postulated, that in human patients spinal osteoblastomas have a predilection site to the posterior elements of the vertebra, including the spinal process, as in the cat presented in this case report, whereas osteosarcomas tend to initially affect the vertebral body. MRI features of spinal osteoblastomas are usually nonspecific and highly variable [47]. Signal intensity in T2-weighted sequences depends on the level of matrix mineralization [48], which is conclusive with the presented case here, that revealed barely mineralized matrix and in turn isointensity to the spinal cord in both T1- and T2-weighted sequences. The well-defined borders of osteoblastomas in CT resemble the histological lack of infiltration at the lesion-host bone interface [38] as seen in the case presented here. OBLOS shares radiological features with both, osteoblastoma and osteosarcoma [19, 44]. Ozger et al. in 2016 [44] postulated, OBLOS to be well- or ill-defined, lytic and sharply demarcated on CT and radiographs. Typical radiographic features of osteosarcoma, like periosteal reaction and/or cortical destruction is a facultative feature in OBLOS. Thinning of the cortex might be present due to the expansile growth [44]. In contrast, OBLOS typically reveals a T1-hypointensity and an intermediate intensity in T2-weighted sequences with heterogeneous contrast enhancement, whereas osteoblastoma shows T1- and T2-iso- to hypointensity with permanent contrast enhancement [44]. Thus, an osteoblastoma at the base of T2 was initially suspected based on radiological examination. On the other hand, in support to the diagnosis of OBLOS, osteoblastoma does not metastasize to other regions of the body [49]. Nevertheless, several reports of osteoblastomas in human medicine, indicate malignant transformation and metastatic behaviour. Bertoni et al. in 1985 [19] described 17 bone tumours, that did not show any permeation of the surrounding bone but in several cases metastatic behaviour. Mitchell and Ackerman in 1986 [28] reported a “*metastatic osteoblastoma*” in a 15-year old boy; Kunze et al. in 1996 [27] and Wozniak et al. in 2010 [29] described metastases following malignant transformation of human osteoblastomas. Metastases have been documented as well in OBLOSs [19, 23]. Interestingly, in all reported cases “osteoblastomas” metastasize into the lungs [26]. The sparsely existing literature indicate that metastatic behaviour of initially diagnosed osteoblastomas have a low incidence with 2 out of 306 (0.7%) in the large cohort study of human patients from Lucas et al. in 1994 [14]. In our feline case, pulmonary metastasis could not be excluded, therefore, without histopathological confirmation we can only speculate that the presented cat suffered from an OBLOS.

Only few reports on osteoblastic tumours have been published in veterinary literature [4, 8–12]. In accordance to the radiological and histopathological findings with special regard to the lung changes and the recurrence, a borderline osteoblastic tumour, sharing most criteria with an OBLOS [19] is a valid diagnosis.

En bloc resection of cervical osteoblastoma has been reported to be the treatment of choice in human medicine [50–52]. Nevertheless, complete excision might be challenging in the vertebral column due to the risk of destabilisation and spinal cord damage. In the presented case, a dorsal laminectomy of the first and second thoracic vertebrae was initially planned. During the removal of the spinous process of T1, the compressive mass was simultaneously detached. In order to avoid destabilization, the dorsal laminectomy was therefore not extended over T2, since the degree of spinal cord decompression was believed to be satisfying. Indeed, the cat improved clinically over the following days. Although strict rest was firmly recommended, the cat was still allowed to run outside. After 3.5 weeks the cat suffered from a sudden, yet transient neurological deterioration. Due to the clinical improvement on conservative treatment, no further work-up was performed. At the follow-up performed 8 months later, a chronic subluxation in the surgery area was diagnosed. It is reasonable to believe that the subluxation happened at the time of the neurological worsening. Although reimaging indicated a recurrence of the mass, the spinal cord was still not compressed. Surgical treatment of spinal bone tumour reported in cats includes an hemilaminectomy [24] in a cervical aggressive osteoblastoma and an hemilaminectomy followed by a vertebral replacement in a L1 osteosarcoma [53]. Recurrence was described two and 3 years after the initial surgery, respectively. In the latter case an additional vertebrectomy was performed 6 months after recurrence and the cat was alive and recurrence-free 5.25 years after the initial hemilaminectomy [53]. Levy et al. in 1997 [54] suspected the difficulty for total excision of vertebral osteosarcoma might be reasonable for the reported better prognosis of cats with appendicular osteosarcoma [3, 55]. Rossmeisl et al. in 2006 [56] found that cytoreductive surgery can achieve a good palliation of clinical signs and spinal cord dysfunction, nevertheless, incomplete excision occurred frequently. The authors found a strong correlation between tumour phenotype and the surgeon’s impression of total resection, since subtotal resection was suspected postoperatively in all malignant and total resection in all benign neoplasms [56]. Based on the intraoperative findings, the whole mass was believed to be resected in the presented case. An important limitation is the lack of immediate postsurgical imaging to assess more objectively the macroscopical amount of tumour tissue removed.

In conclusion and in respect to the entire radiological, clinical and pathological findings, the vertebral neoplasia in the reported cat, best resembles what in human medicine is mainly known as OBLOS [19] and it represents the first description in a feline patient.

Our case report depicts the challenges in diagnosing primary bone tumours and gives a wide overview about the current discussion on their definitions and classifications. The primary bone neoplasia in this cat clearly compounds features of both osteoblastoma and osteosarcoma and thus might represent a novel subtype of primary bone tumours. Additionally, it contributes interesting features to the sparsely existing literature on feline primary bone tumours. Malignant transformation of an osteoblastoma have not been described in veterinary medicine yet. This case report also reflects the almost daily clinical challenge in estimating the dignity of a lesion, which is in the end more important than the correct naming.

## Data Availability

All data generated or analysed during this study are included in this published article.

## Abbreviations

COX-2: Cyclooxygenase-2
CT: Computer tomography
EDTA: Ethylenediaminetetraacetic acid
HU: Hounsfield Unit
MRI: Magnet resonance imaging
OBLOS: Osteoblastoma-like osteosarcoma
STIR: Short tau inversion recovery
